# Molecular Mechanisms of p63-Mediated Squamous Cancer Pathogenesis

**DOI:** 10.3390/ijms20143590

**Published:** 2019-07-23

**Authors:** Michael A. Moses, Andrea L. George, Nozomi Sakakibara, Kanwal Mahmood, Roshini M. Ponnamperuma, Kathryn E. King, Wendy C. Weinberg

**Affiliations:** Laboratory of Molecular Oncology, Office of Biotechnology Products, Center for Drug Evaluation and Research, Food and Drug Administration, 10903 New Hampshire Avenue, Silver Spring, MD 20993, USA

**Keywords:** p63, p53 family, keratinocytes, squamous carcinogenesis, epidermal homeostasis, epidermal morphogenesis

## Abstract

The *p63* gene is a member of the p53/p63/p73 family of transcription factors and plays a critical role in development and homeostasis of squamous epithelium. *p63* is transcribed as multiple isoforms; ΔNp63α, the predominant p63 isoform in stratified squamous epithelium, is localized to the basal cells and is overexpressed in squamous cell cancers of multiple organ sites, including skin, head and neck, and lung. Further, p63 is considered a stem cell marker, and within the epidermis, ΔNp63α directs lineage commitment. ΔNp63α has been implicated in numerous processes of skin biology that impact normal epidermal homeostasis and can contribute to squamous cancer pathogenesis by supporting proliferation and survival with roles in blocking terminal differentiation, apoptosis, and senescence, and influencing adhesion and migration. ΔNp63α overexpression may also influence the tissue microenvironment through remodeling of the extracellular matrix and vasculature, as well as by enhancing cytokine and chemokine secretion to recruit pro-inflammatory infiltrate. This review focuses on the role of ΔNp63α in normal epidermal biology and how dysregulation can contribute to cutaneous squamous cancer development, drawing from knowledge also gained by squamous cancers from other organ sites that share p63 overexpression as a defining feature.

## 1. Introduction to the *p53/p63/p73* Gene Family of Transcription Factors 

The importance of p53 in the maintenance of genomic integrity is underscored by the observation that mutation or inactivation of p53 is a common event in human cancers. Almost two decades after the p53 gene was described, two additional family members, p63 and p73, were identified based on structural similarities in the major p53 functional domains: the transactivation (TAD), DNA binding (DBD), and oligomerization (OD) domains [1,2]. In contrast to the classical understanding of p53, these new family members were shown to consist of multiple protein isoforms resulting from alternate promoter usage and C-terminal splicing. p63 and p73 both include two subclasses of proteins containing either TA or ΔN domains at the amino terminus. The TAp63 isoforms contain a p53-like consensus transactivation domain that can mimic p53 function, while the ΔN isoforms lack this domain and act as dominant negatives to TAp63/73, as well as to p53. Despite the lack of a TA domain, the ΔN isoforms of p63 can positively regulate gene transcription through additional transactivation domains [3,4,5,6]. Shortly after the discovery of the p63 and p73 isoforms, similarly transcribed p53 isoforms were described that are co-expressed with canonical p53, adding additional biological complexity that can influence functional outcome [7].

**Figure 1:**
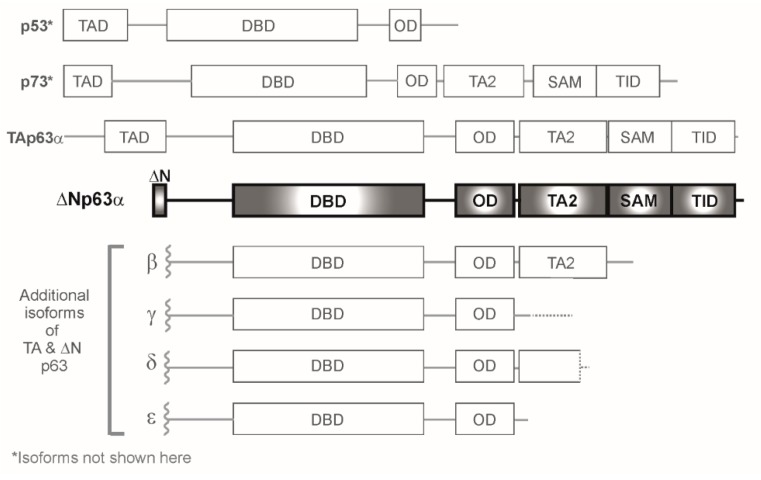
Schematic of p53/p63/p73 family members. Within the p63 homologues, alternative promoter usage yields TAp63 and ΔNp63 subclasses; within each subclass, alternative spicing yields α, β, γ, δ, and ε isoforms. This review focuses on ΔNp63α (highlighted), the predominant isoform in squamous epithelium. Domain abbreviations: TAD—Transactivation domain, ΔN—Delta N Domain, DBD—DNA binding domain, OD—Oligomerization domain, TA2—Transactivation domain 2, SAM—Sterile alpha motif, TID—Transactivation-inhibitory domain.

Between the two subclasses of p63, a total of ten p63 isoforms arising from C-terminal alternative splicing have been described to date: TA- and ΔN- p63α, β, γ, δ, and ε [1,8] (Figure 1). Structurally, the C-terminus of ΔNp63α contains further functional protein domains including a Sterile Alpha Motif (SAM) protein–protein interaction domain, a transactivation inhibitory domain (TID) [9,10], and two distinct alternate transactivation domains: one named TA2, encoded by exon11 and 12 [4], and another in the ΔN terminus [3,6]. Beyond p63, twenty-nine p73 mRNA transcripts, which may not all be translated, and twelve p53 protein isoforms have been described [7,11]. The p53 family members function as tetramers through their oligomerization domains, with p63 and p73 preferentially interacting with one another, rather than with p53, and heterotetramers being the preferred configuration [12]. p63/p73 interactions with p53 have been demonstrated to occur through the DBD; wild type (WT) p53 targets ΔNp63α for degradation through this domain [13], while mutant p53 also interacts with p63 and p73 through this domain, thus impairing their transactivation capacities [14,15]. As such, the structural similarities between the p53/p63/p73 family members allow them to interact with one another through a variety of mechanisms. Therefore, the balance of the isoforms in a given context, as well as their relative expression levels, can ultimately impact biological outcome.

While canonical p53 is ubiquitously expressed and activated upon cellular stress, p63 and p73 isoforms exhibit tissue-specific expression patterns and play critical roles in normal development and homeostasis [16,17]. ΔNp63α is the predominant isoform present in adult human epidermis and its expression is associated with the proliferative compartment of the skin [16]. In vivo models revealed that *p63* is essential for normal epidermal development and homeostasis [18,19,20], and in humans, *p63* mutations have been associated with ectodermal dysplasia syndromes that include skin phenotypes [21]. Likewise, *p73* demonstrates tissue-specific roles, as its loss has been linked to abnormalities in development of the nervous system and ciliogenesis [22,23]. Initially, there was anticipation that mutations in p63 might contribute to the development of cancers in which p53 is not mutated; however, rather than mutation, overexpression of p63 and especially the ΔNp63 isoforms has been associated with malignant conditions including squamous carcinomas, such as those of the head and neck and skin [24,25,26]. This review provides an overview of the critical role of p63, particularly ΔNp63α, in normal epidermal development and homeostasis, with an emphasis on the multiple pathways impacted by ΔNp63α dysregulation that are implicated in squamous cancer pathogenesis.

## 2. Mechanisms of Transcriptional Regulation by p63

**Figure 2:**
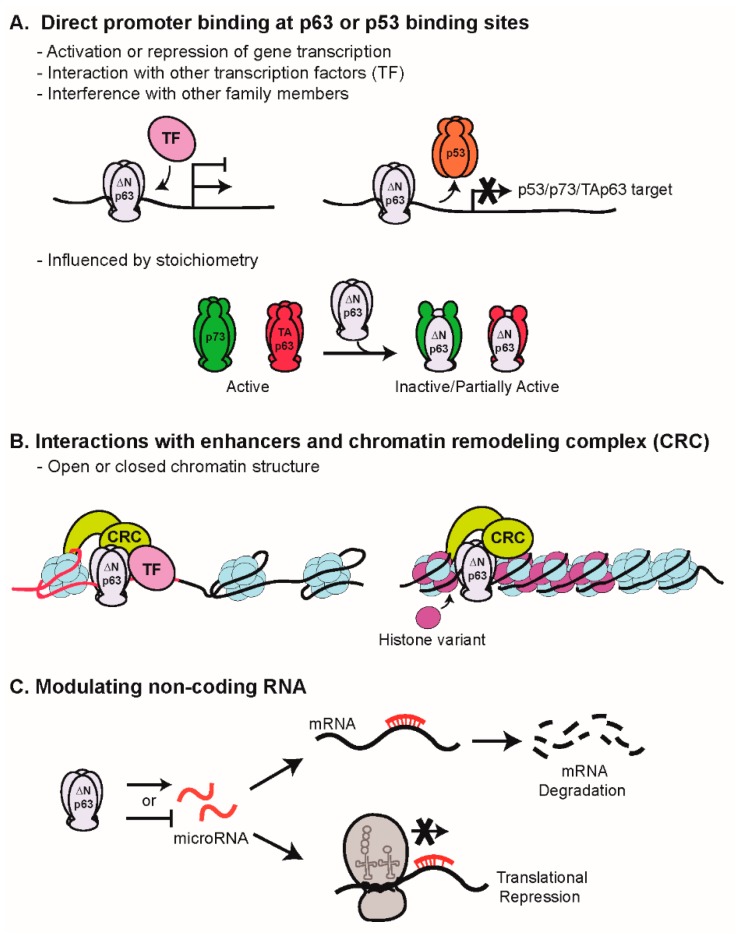
p63 mediates gene expression via multiple mechanisms. (**A**) Direct promoter binding: ΔNp63α activates or represses transcription by binding to promoters of its target genes and associating with other transcription factors to enhance or suppress transcriptional activity. The stoichiometric balance of the p63 and p73 isoforms can modulate the activity; these interactions can lead to different transcriptional programs and altered biological outcomes. (**B**) Interactions with enhancers and chromatin remodeling complex: At sites of high histone acetylation (e.g.; H3K27), p63 interaction with the chromatin remodeling complex (CRC) maintains an open chromatin landscape controlling access to epidermal enhancers and super enhancers to drive gene activation in conjunction with other transcription factors. In this way, p63 directs tissue-specific gene regulation guiding epidermal differentiation. (**C**) Activation or repression of non-coding RNAs: Long non-coding RNA and microRNA are targets of ΔNp63 regulation, thereby indirectly modulating gene expression. Examples of ΔNp63α activating or repressing transcription of miRNAs are shown.

p63 impacts gene expression profiles both locally and globally through multiple mechanisms (Figure 2). These include direct binding of gene promoters, “bookmarking” of enhancers and defining the chromatin landscape (open vs. closed) in a context-specific manner, and regulation of non-coding RNAs.

Due to the shared homology within the DBD of the family members, DNA binding is an area in which the balance of the isoforms is crucially important (Figure 2A). The ∆Np63 isoforms are capable of binding to canonical p53 DNA binding sites and thus compete with p53 and TAp63/73 [1]; however, discrete p63 consensus binding sites have also been described [27,28,29]. ΔNp63α can both activate and repress gene transcription [3,4,6]. Using a genome-wide mapping approach, p63 and p73 were found to share genomic targets in a cervical carcinoma cell line in vivo, which could translate into a biological outcome influenced by relative expression levels of the isoforms present in a given cellular context [30]. Context-dependent co-factors also impact direct gene regulation. ΔNp63α has been shown to physically interact with transcription factors, such as SOX2, a stem cell-associated transcription factor [31], c-Rel, a member of the NF-κB family [32], and Y-box binding protein-1, a nucleic acid binding protein involved in multiple DNA/RNA-dependent processes [33]. Cooperation of each of these factors with ΔNp63α has been linked to survival and/or proliferation.

p63 can also influence gene expression in a global manner through chromatin remodeling (Figure 2B). In an epigenomic-profiling exercise, p63 binding was characterized at enhancer sites during epidermal differentiation, and unexpectedly, the p63 binding pattern remained relatively unchanged over the course of this process [34]. Approximately half of the p63 binding sites co-marked with H3K27ac, a marker of activity, and this correlated with expression of nearby genes, suggesting that p63 can serve as a “bookmark” for genomic loci in the epithelial lineage that may then be activated by additional transcriptional factors [34]. In addition, it was demonstrated that p63 and the catalytic subunits of the BAF (SWI/SNF) chromatin remodeling complex (Brg1 or BRM) are required to maintain a cell type-specific open chromatin landscape controlling epidermal enhancers during differentiation; this relationship was not preserved in cells lacking p63 [35]. Mechanistically, in the presence of p63, BAF displaces nucleosomes around p63 binding sites and recruits transcriptional machinery [35]. In contrast, a SWI/SNF subunit, ACTL6A, physically associates with ΔNp63α on regulatory elements to decrease chromatin accessibility, resulting in altered gene transcriptional profiles in a subset of head and neck squamous cell carcinomas (HNSCCs) [36]. 

Non-coding RNAs, such as microRNAs (miRNAs) and long non-coding RNAs (lncRNAs), are alternate mechanisms whereby gene transcription can be indirectly regulated by p63 (Figure 2C). miRNAs (approximately 19–22 nucleotides) recognize and bind the 3’-UTR sequences of target mRNA, thereby inducing degradation and/or preventing translation, and can either be activated or repressed by p63 or other regulators. miRNAs targeted by p63 can impact expression of both epigenetic regulatory factors, as well as direct transcriptional regulators, to alter keratinocyte growth regulation and response to chemotherapeutics [37,38,39,40]. LncRNAs are polyadenylated RNAs greater than 200 nucleotides that do not have an open reading frame; they modulate gene expression via wide-ranging mechanisms involving both transcriptional and post-transcriptional regulation [41]. LncRNAs are tightly regulated in normal biology, but are dysregulated in cancer, including HNSCC [42,43], and have recently been identified as ΔNp63α targets [44]. 

Although this review focuses on ΔNp63α-dependent regulation of downstream signaling networks and effectors, the regulation of ΔNp63α is an additional point worth noting. While not covered exhaustively in this review, mechanisms including, but not limited to, stabilization, degradation, and cellular localization are involved in modulating ΔNp63α activity (reviewed in [45]). Recently described examples of additional mechanisms include interactions with syntaxin-binding protein 4 to suppress the proteolysis of ΔNp63α, leading to decreased turnover [46,47], and increased activity of the nucleoporin NUP62 to facilitate ΔNp63α nuclear import, potentiating its role as a transcription factor [48,49]. Differential methylation status of the ΔNp63α gene locus is also involved in driving its expression [26]. In the following sections, we discuss the multifaceted roles of ΔNp63α in development, tissue homeostasis, and cancer pathogenesis.

## 3. ΔNp63α Is Essential for Normal Morphogenesis and Squamous Epithelial Homeostasis

Early studies of *p63^−/−^* mice revealed a dramatic absence of stratified squamous epithelium, which suggested roles for p63 in lineage commitment and/or stem cell maintenance [18,19]. Following these initial observations, multiple groups created models to explore the impact of gain or loss of a single p63 isoform. Approaches included basal cell-targeted overexpression, tissue-specific knock-in (on a *p63*-null background), and isoform-specific knock-out transgenic lines. In one model, TAp63α overexpression driven by the keratin 14 promoter in WT mice resulted in a hyperplastic epidermis and loss of terminal differentiation, suggesting that the TAp63α isoform is responsible for driving epithelial stratification [50]. However, knock-in studies on a *p63*-null background failed to reflect these effects, instead revealing that reconstitution of TAp63α in keratin 5 expressing keratinocytes was insufficient to generate a complete epidermis [51]. In contrast to these TAp63α studies, keratin 5-driven expression of ΔNp63α in *p63^−/−^* mice was shown to partially restore the epidermal basal layer and expression of keratins 5 and 14, but not upper epidermal layer markers keratin 1 or loricrin [51]. Similarly, in a separate model, induction of ΔNp63α or ΔNp63β by the keratin 5 promoter in a *p63*-null background partially restored epithelial integrity, stratification, and expression of differentiation markers, leading to the conclusion that ΔNp63α or ΔNp63β can initiate stratification [52]. In agreement with a role for ΔNp63α in regulating epidermal development and commitment, specific knockout of ΔNp63α isoforms largely reiterated the phenotype of original *p63*-null mice [53] (summarized in Table 1). Taken together, these results suggest that a finely tuned balance of isoforms is required for complete epidermal stratification. In further support, a greater degree of structured epidermal formation and differentiation was observed in mice expressing both TAp63α and ΔNp63α with expression of keratins 1, 5, 14, and loricrin, compared to single isoform reconstitution [51].

Increasing evidence has supported a role for p63 in stem cell maintenance [54,55,56]. The high replicative potential of stem cells, along with studies suggesting progenitor cell exhaustion and non-regenerative differentiation in *p63*-deficient mice [18,53], further supports the hypothesis that p63 is involved in maintaining epithelial stemness and regenerative capacity. Within the epidermis, ΔNp63α is highly expressed in stem cells [55] and basal keratinocytes of stratified and glandular epithelial compartments [54,55,56]. Utilizing two independent lines of *p63*-deficient mice, evidence of premature aging was observed in *p63^+/−^* mice [57,58] and attributed to diminished progenitor cell self-renewing capacity. Both germline and keratin 5 promoter-mediated somatic depletion of *p63* in keratinocytes led to increased expression of the senescence markers senescence-associated β-galactosidase (SA-β-gal), p16^INK4A^, and promyelocytic leukemia protein, suggesting stem cell exhaustion associated with the aging process [58]. Furthermore, epidermal-specific conditional knockout of TAp63 in transgenic mice led to premature senescence, depleted precursor cell populations, and premature skin aging [59]. A stem cell role for ΔNp63α was also suggested by the observation of a 2-fold increase in the number of keratinocytes expressing CD34^+^, a marker of keratinocyte stem cells, in epidermal cells isolated from keratin 5-driven ΔNp63α transgenic mice relative to WT controls [60]. Collectively, these data support the notion that p63 isoform activity is involved in promoting stem cell maintenance and tissue renewal. 

In addition to the role played by p63 as a transcription factor that directly targets genes impacting normal skin development, it has become apparent that p63 plays a wider role as a regulator of epidermal cell fate via epigenetic regulatory mechanisms. The transcription factor TFAP2C and its interplay with p63 in epidermal development was identified in a recent study using cell culture models to define changes in the chromatin landscape as cells transition from pluripotent stem cells through surface ectoderm progenitor cells to become mature keratinocytes [61]. In committed epithelial cells, TFAP2C prepares the chromatin landscape for ΔNp63α-dependent generation of stratified epidermis by activating p63 expression and by increasing chromatin accessibility surrounding p63 binding sites to prime keratinocyte maturation. Feedback regulation between p63 and TFAP2C enforces this epidermal lineage maturation; with increasing levels, p63 begins to function independently of TFAP2C and self-regulate its own expression, leading to the closing of TFAP2C binding sites that are associated with ectoderm progenitor cells. As such, TFAP2C is indispensable for the transition from pluripotent stem cells to surface ectoderm commitment (expressing keratins 8 and 18) [61]. In contrast, ΔNp63α interactions with chromatin modifiers can mediate transcriptional repression. Histone deacetylases (HDACs) allow DNA to wrap more tightly around histone proteins following their deacetylation to limit gene transcription. Mice generated with keratin 14-driven deletion of both *HDAC1* and *HDAC2* present with a phenotype reminiscent of the *p63^−/−^* mice. Mechanistically, HDAC1/2 were determined to be necessary for the negative regulation of gene targets repressed by ΔNp63 (including the cell cycle regulators p16^INK4A^ and p21^WAF1^) in undifferentiated cells, but did not affect positively regulated basal cell targets [62]. 

**Figure 3:**
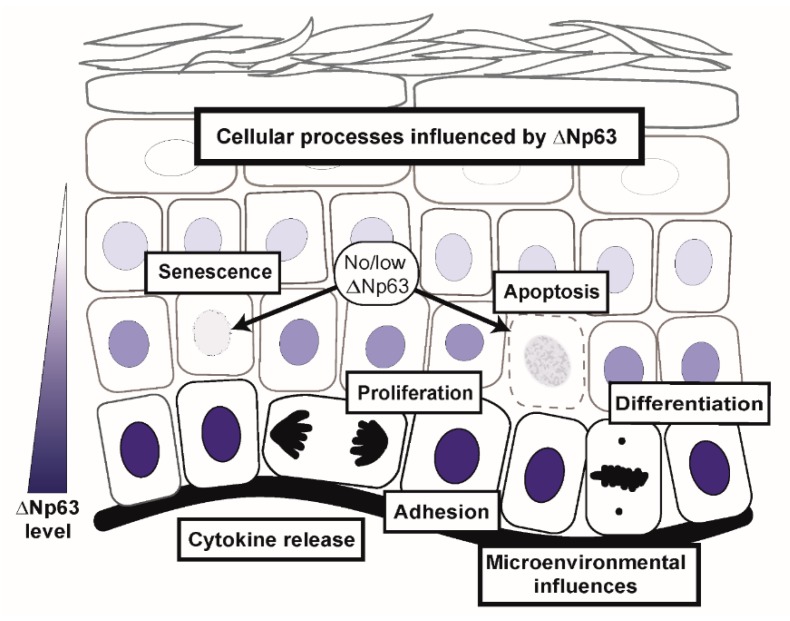
ΔNp63α regulates cell intrinsic and extrinsic biological processes involved in normal epidermal morphogenesis and homeostasis. In cancer, amplification of *p63* leads to the hijacking of these processes to support conversion to and progression of the malignant state.

Once tissue is established, epidermal homeostasis requires a balance between positive and negative growth influences, which may include altered proliferation signaling and induction of differentiation, senescence, and apoptosis (Figure 3), all of which are under the influence of ΔNp63α and its activation/repression of downstream targets [63]. These include (but are not limited to) *bone morphogenetic protein (BMP) 7* (through direct binding to the *BMP7* promoter [64] or direct repression of *Smad7* [65]), Notch1 [66,67], *Dlx3* [21,68], sonic hedgehog (SHH, through activation of *SUFU* [69]), *keratin 14* [70], *fibroblast growth factor receptor 2* (*FGFR2*) [64,71,72], and *transforming growth factor* β (*TGF-*β, [73]). More recently, it was reported that ΔNp63α supports epidermal differentiation by binding to the *ZNF185* enhancer and increasing its expression. ZNF185 co-localizes with E-cadherin in cadherin junctions during epithelia stratification and differentiation [74]. Other studies have focused on the role of p63 in the regulation of the epidermal differentiation complex (EDC). Microarray analysis of WT and *p63*-null E16.5 epidermal progenitor cells revealed enrichment in genes in WT samples for chromatin and nuclear assembly factors including *Satb1* [75] and *Brg1* [76], both of which are direct p63 targets. Ablation of either *p63* or *Satb1* altered the chromatin conformation at the center of the EDC domain and was associated with a reduction in expression of genes associated with epidermal barrier function located in this region [75]. In addition, Brg1 was shown to be required for relocation of the EDC to the nuclear interior [76]. KMT2D, a histone methyltransferase, also interacts with p63 at target enhancers to maintain adhesion and proliferative capacity in normal epidermal homeostasis [77].

### p63 Mutations Are Associated with Human Ectodermal Dysplasia Syndromes

The importance of p63 (and its target genes) in development and epidermal homeostasis is underscored by the association of human germline mutations in *p63* with developmental disorders characterized by ectodermal dysplasias, including limb truncations, craniofacial malformations, and dysregulation of the developing epidermis. The ectodermal dysplasia syndromes are linked to distinct domains of the p63 gene and display varying levels of involvement of the epidermis and associated appendages [21]. Mutations in the DNA binding domain have been implicated in Ectrodactyly Ectodermal Dysplasia-Clefting Syndrome (EEC) and abrogate p63 transcriptional activity [78]. Two additional syndromes associated with p63 DNA binding domain mutations are Limb-Mammary Syndrome and Acro-Dermato-Ungual-Lacrimal-Tooth Syndrome [79,80]. In contrast, Ankyloblepharon-Ectodermal Dysplasia Clefting syndrome (AEC) is linked to a heterozygous missense mutation in the SAM region of p63 [81]. Such mutations have been reported to prevent interactions between ΔNp63α and RNA-splicing machinery critical for correct splicing of FGFR2 to the isoform responsible for normal epithelial differentiation [82]. Mutations in the p63 SAM domain that cause AEC have also been shown to lead to reduced transcription of *Dlx3* [83], a homeobox transcription factor involved in keratinocyte terminal differentiation. 

Corroborating the role of p63 in control of chromatin remodeling, a global alteration in the transcriptional regulatory program of normal epidermal genes that are markers of epidermal cell identity was observed across patient-derived EEC mutant keratinocytes relative to controls. This can be explained by loss of the dynamic association of p63 with its regulated enhancers during normal differentiation, as discussed above [34]. These findings further underscore the criticality of p63-regulated gene networks in normal epidermal morphogenesis and differentiation [84].

## 4. Dysregulated ΔNp63α Disrupts an Extensive Network of Molecular and Biological Pathways to Contribute to Squamous Cancer Pathogenesis

Accumulating evidence demonstrates that human squamous cell carcinomas (SCCs) from different organs share common activated pathways [26,85,86]. One of the most common genomic alterations shared between SCC of lung, head and neck, esophagus, cervix, and bladder is amplification of the chromosome region between 3q26 and 3q28, which includes *p63*; this is associated with a predominance of mRNA for the ΔNp63α isoforms relative to TAp63 mRNAs [26]. Similarly, according to data sets found in the cBioportal for Cancer Genomics (cbioportal.org), *p63* is altered or amplified in 47% of patients/samples with lung SCC (The Cancer Genome Atlas, TCGA, provisional, *n* = 178 patients/samples), and in 23% of patients/samples with HNSCC (TCGA, provisional, *n* = 504 patients/samples) [87,88]. These data are consistent with noted properties of ΔNp63α in epithelial proliferation, as described above. While not included in the cross-tissue SCC study [26], an examination of genomic alterations of metastatic cutaneous squamous cell carcinoma (cSCC) in 29 patients identified amplification of WT *p63* in 24% of samples [25]. Thus, information from SCCs derived from multiple organ sites can be leveraged to expand our understanding of p63 function and will be discussed in the following sections.

### 4.1. ΔNp63α Mediates Signaling Pathways Impacting Multiple Cell Intrinsic Biological Processes

*p63* amplification and overexpression, frequently observed in SCCs, has also been shown to result in a unique gene expression profile compared to basal levels of ΔNp63α expressed in normal cells. In a recent study by Saladi et al. [36] correlating chromatin immunoprecipitation-sequencing (ChIP-Seq) and microarray analyses, ΔNp63α binding sites in the genome were compared between normal keratinocytes and SCC cell lines (JHU029, HCC95, TT, FaDu). Among differentially regulated pathways, ΔNp63α modulates integrin-mediated cell adhesion, epidermal growth factor receptor (EGFR) 1 signaling, mitogen-activated protein kinase (MAPK), and T-cell receptor signaling pathways in tumor-derived cell lines, while senescence/autophagy, glutathione metabolism, and insulin signaling pathways are impacted in normal keratinocytes [36]. These data indicate context-dependent transcriptional regulation by ΔNp63α.

In normal stratified squamous epithelium, nuclear p63 expression is predominantly localized in the basal proliferative compartment with a reduction in expression in the more superficial layers. In SCC, a stronger nuclear and more tissue diffuse pattern has been observed, where increased p63 levels extend throughout the tumor tissue [16,32,89]. Numerous groups have developed both in vitro and in vivo models to mimic the overexpression of ΔNp63α observed in human cancers to determine if it has a contributory role in cancer pathogenesis (Table 1). In a murine orthotopic grafting model of multistage carcinogenesis using primary mouse keratinocytes, elevated ΔNp63α was shown to cooperate with the oncogenic H-Ras pathway to drive malignant progression of H-Ras-initiated tumors [90]. Furthermore, overexpression of ΔNp63α in this model promoted cell survival and inhibited both cellular replicative and oncogene-induced senescence, as evident by cellular morphology, SA-β-gal staining, and reduced p16^INK4A^ and p19^ARF^ levels. This activity resides, at least in part, in the ability of ΔNp63α to directly bind to the p16^INK4A^ and p19^ARF^ promoters to repress their expression and prevent cellular senescence [90,91]. In this regard, it is notable that crossing mice lacking all p63 isoforms [18] with mice devoid of p16^INK4A^ or p19^ARF^ leads to a partial restoration of keratinocyte proliferation and differentiation [91]. The cooperation between ΔNp63α and oncogenic Ras is also consistent with results from Keyes et al. [92] in which ΔNp63α overexpression bypasses senescence via induction of the chromatin remodeler, Lsh [92]. In addition to these pathways, ectopic expression of ΔNp63α was shown to bypass cellular senescence by preventing p38 MAPK phosphorylation via upregulation of MAPK phosphatase 3 [93], thus preventing activation of p53 and subsequent cell cycle arrest. In further support for a role in promoting cell survival, the physical association of ΔNp63α with HDAC1/2 prevents apoptosis in SCC by suppressing *PUMA*, a pro-apoptotic gene [94].

In mouse models of carcinogen-induced (DMBA) cutaneous and oral SCC, ΔNp63α was demonstrated to be overexpressed and to play an indispensable role in tumor progression. Following the establishment of carcinogen-induced tumors, conditional deletion of *p63* in *p53*-deficient transgenic mice (*p63^L/L^K14-CreER/p53^+/−^*) by tamoxifen resulted in rapid regression of both cutaneous and oral tumors [95]. Consistent with the role of ΔNp63α as a regulator of FGFR2 [64], further analyses revealed the fibroblast growth factor (FGF) signaling pathway, specifically FGFR2, was shown to be significantly upregulated by ΔNp63α and required for tumor progression to occur in this model [95]. In mice with moderate, tissue-specific expression of ΔNp63α driven by the keratin 5 promoter, expression of the proliferation marker, Ki-67, and differentiation markers, keratin 10 and loricrin, were comparable between the skin of WT and ΔNp63α-transgenic mice [60]. This indicates that moderate expression of ΔNp63α does not significantly alter the basal phenotype. However, the ΔNp63α mice are more susceptible to mutagen-induced tumor initiation and progression, and in vitro analyses demonstrated a delay in senescence with increased p53, Sirt1, and Lsh, and suppressed p16^INK4A^ and p19^ARF^ levels [60], consistent with established roles of ΔNp63α as a contributor to carcinogenesis [90,92].

∆Np63α may also play a major role in controlling epithelial-to-mesenchymal transition (EMT) through maintenance of the epithelial phenotype; however, its exact role remains controversial. ∆Np63α overexpression was reported to restrict EMT in a skin model of well-differentiated SCC in which ∆Np63α binding sites were associated with open chromatin, compared to keratinocyte-derived tumors with a more mesenchymal phenotype [96]. This supports a role for ∆Np63α in maintaining a chromatin landscape which directs an epithelial phenotype [34,35]. In SCC and normal keratinocytes, ∆Np63α upregulates epithelial genes such as claudin 1 [97], an epithelial cell marker, and integrins (β1 and α6) involved in cell adhesion [98]. Consistent with these findings, overexpression of ∆Np63α in prostate epithelial cells led to global gene expression changes favoring the epithelial phenotype, including enrichment of cell signaling pathways involved in cell–cell adhesion and interactions with the extracellular matrix [99]. An EMT suppressive role by ∆Np63α has also been reported in bladder cancer cells and mammary cells; following knockdown of ∆Np63α, these effects were shown to be dictated by decreased levels of ∆Np63α-activated epithelial-specific miRNAs (i.e.; miRNA-205) [100,101].

In contrast, ΔNp63α modulates Wnt, Notch, BMP, and other TGF-β signaling pathways, whose downstream effectors (i.e.; Snail, Slug, Twist) are transcriptional regulators of genes involved in cell adhesion and migration that promote the EMT phenotype. These pathways and EMT-inducing effectors can also exist in a negative feedback loop to inhibit ∆Np63α activity and enhance EMT [102]. In the context of wound healing, ΔNp63α upregulates the TGF-β pathway through activation of its effectors, SMAD4 and TGF-βR2, thereby facilitating the EMT features of invasion and motility [103]. In addition, silencing of the p63-regulated chromatin organizer *Satb1* in SCC cells has also been shown to reverse the expression of EMT markers [104]. In another example of the importance of the relative balance of p63 isoforms, ∆Np63γ, but not ∆Np63α, can promote EMT in association with SRC-dependent transcription of Slug in HNSCC cells [105]. Overall, based on these observations, it is likely that ∆Np63α plays a critical role in EMT depending on the cell type, stage of cancer, and balance of other p63 isoforms.

∆Np63α modulates additional intrinsic signaling through interactions with non-coding RNA elements. The inhibitor of apoptosis-stimulating protein of p53 (iASPP)-p63 feedback loop, whereby p63 positively regulates iASPP (an anti-apoptotic gene) at the mRNA and protein levels (and iASPP positively modulates p63 only at the protein level) via the repression of miRNAs-574-3p and -720, was previously shown in normal epidermal homeostasis to sustain proliferation and adhesion [106]; it has also has been linked to cSCC [107]. Furthermore, bioinformatics analyses identified a number of miRNAs that target p63, a large percentage of which are also predicted to be regulated by p63 in a cross-talk mechanism [108]. miRNAs targeted by p63 can impact expression of both epigenetic regulatory factors, as well as direct gene transcriptional targets that can impact chemosensitivity [38,40]. In addition to the FGF growth factor receptor described previously, ΔNp63α also plays a role in modulating EGFR activity via non-coding RNAs. In SCC cell lines, p63 and SOX2 were found to co-occupy super enhancers for more than 50 transcripts, but not typical enhancer sites [109]. One target transcript bound both at its promoter and at its super-enhancers by p63/SOX2 was the lncRNA *CCAT1* [109], which has previously been associated with malignancy [110]. Expressed CCAT1 forms a complex with p63 and SOX2 on super enhancers for EGFR resulting in sustained, dysregulated EGFR expression and activation of its downstream signaling pathways associated with growth and proliferation [109].

#### 4.1.1. p63 and Cancer Stem Cells

Efforts have been predicated on the concept that cancers initiate from progenitor, or cancer stem cells, which reside in a specialized tumor niche and are capable of giving rise to heterogenous cell progeny. This concept is derived from the observation that tumor cells often display characteristics similar to those of stem cells, including resistance to apoptosis, senescence, and drug therapies [111,112]. Defining these specific cancer stem cell (CSC) populations has remained controversial and identifying markers for these resistant CSC populations presents a goal toward developing targeted therapies. Multiple CSC markers have been proposed for squamous cell carcinomas, including the cell surface markers CD44, CD133, and “stem cell” signaling proteins SOX2, MYC, and p63 [113,114,115,116]. Ripamonti et al. [117] demonstrated that treatment of SCC cell lines with epithelial growth factor led to an increase in ΔNp63α expression and tumor initiating cell proliferation, indicating the importance of ΔNp63α in SCC tumor stem cell maintenance [117]. In SCC, *SOX2* is co-amplified with *p63* [26] and preferentially interacts with ΔNp63α to regulate the oncogene ETV4, leading to SCC cell proliferation [31]. Additionally, the gene for the chromatin remodeling factor *ACTL6A* (a SWI/SNF subunit gene) has been shown to be co-amplified with *p63* in HNSCC. Concomitant upregulation of p63/ACTL6A results in an enhancement of the stem-like regenerative gene transcription program and inhibition of terminal differentiation [36]. ACTL6A activity also leads to sequestration of differentiation-promoting chromatin modifiers, thereby promoting more “stem-like” characteristics [118]. This appears to be orchestrated through cooperation between p63 and ACTL6A to decrease chromatin accessibility, leading to repression of *WWC1* and activation of YAP, a key oncogenic downstream effector in the Hippo pathway involved with stem cell self-renewal and identity [36,119]. In addition, YAP1 can interact with and stabilize ΔNp63α to promote cancer stem cell survival [120]. Interestingly, SOX2 was shown to suppress the Hippo pathway by inhibiting WWC1 and NF2 in osteosarcoma cell lines and NIH-3T3 fibroblasts, resulting in YAP1 activation [121]. While this pathway has not been delineated in epithelial cells, SOX2 can interact with p63 in the same loci to co-regulate genes [31], suggesting a role for ΔNp63α as one of the centerpieces of interactions to dysregulate the Hippo pathway and activate YAP to maintain stem-cell like properties.

#### 4.1.2. p63 and Cellular Metabolism

The increased proliferative potential of epidermal precursor cells necessary to sustain the development and regeneration of the epidermis is a highly energetic process. Elevated proliferative capacity is also a hallmark of cancer cells, which rely on increased glycolysis and aerobic respiration to generate ATP to support the malignant phenotype. These diverse, yet overlapping, metabolic pathways including glucose and lipid metabolism, as well as oxidative phosphorylation, have been shown to be regulated by TAp63, ΔNp63α, or their transcriptional targets. 

In one of the first studies describing a link between TAp63 and metabolism [122], using isoform-specific knock-out mice, loss of TAp63 (with ΔNp63α expressed) was shown to lead to defects in glucose and lipid metabolism. Specifically, *TAp63*-deficient mice were characterized by obesity, insulin resistance, glucose intolerance, reduced glucose uptake, increased fatty acid synthesis, and reduced fatty acid oxidation resulting in lipid accumulation in the blood and liver. Elucidation of the mechanism for these effects revealed that TAp63 transcriptionally regulates several genes involved in glucose and lipid metabolism including *Sirt1*, *AMP-activated protein kinase α2*, and *LKB1* to ensure metabolic homeostasis [122].

Other studies have demonstrated a role for the ΔNp63α isoform in regulating glycolysis and/or mitochondrial metabolism. In human neonatal foreskin keratinocytes, knockdown of ΔNp63α with siRNA impaired glycolytic activity due to a reduction in the p63 target gene *6-phosphofructo-2-kinase/fructose-2,6-bisphosphatase 3* [123]. In contrast, loss of ΔNp63α in primary human keratinocytes reduced oxidative phosphorylation and increased mitochondrial oxidative stress and membrane hyperpolarization. These effects were mediated through direct ΔNp63α transcriptional regulation of *hexokinase 2* (*HK2*) [124]. HK2, a glycolytic enzyme involved in glucose metabolism, interacts with the mitochondrial voltage-dependent anion channel [125] and couples oxidative phosphorylation to glycolysis for efficient energy production and ADP/ATP recycling. Loss of ΔNp63α results in diminished HK2 expression, thereby disrupting ATP production and mitochondrial membrane potential and causing an imbalance in the proton pump gradient. This imbalance not only leads to decreased oxygen consumption and impaired glycolysis, but results in increased reactive oxygen species (ROS) generation. However, ΔNp63α also plays an antioxidant role, as the levels of enzymes involved in combating oxidative stress, including glutathione peroxidase 2 (GPX2), mitochondrial superoxide dismutase, and NADPH quinone oxidoreductase, are reduced when ΔNp63α is silenced [124]. Indeed, *GPX2* itself has been shown to be a direct target gene of ΔNp63α and protects MCF7 breast cancer cells against oxidative stress [126]. Furthermore, in human primary keratinocytes, ΔNp63α transcriptionally regulates *cytoglobin*, a ROS scavenger that monitors oxygen concentration in the mitochondria. Not surprisingly, cytoglobin also has a protective role in cancer cells and proliferating keratinocytes, and its interaction with ΔNp63α may be clinically relevant in lung cancer patients [127].

Collectively, these data demonstrate distinct roles for both TAp63 and ΔNp63α (and their target genes) in maintaining cellular metabolic homeostasis, whereby TAp63 controls glucose and lipid metabolism and ΔNp63α regulates glycolytic effector proteins and couples glycolysis to oxidative phosphorylation. It is likely their particular metabolic functions are cell, tissue, and/or tumor-type specific; nonetheless, these metabolic observations suggest a role for p63 in regulating energy demands essential for malignant transformation and progression.

### 4.2. ΔNp63α Modulates Signaling Pathways Influencing the Extracellular Microenvironment

In addition to mediating intrinsic cellular properties, ΔNp63α may influence the extracellular microenvironment in a manner that facilitates tumor migration and metastases. In HNSCC cell lines, ΔNp63α induced expression of hyaluronic acid, a major component of the extracellular matrix [128]. In primary murine keratinocytes, elevated levels of ΔNp63α resulted in downregulation of protease inhibitors including maspin (serpinB5), plasminogen activator inhibitor-2 (PAI-2; serpinB2), and tissue inhibitor of metalloproteinase (TIMP)-3, observed at both the RNA and protein levels. These protease inhibitors are associated with maintaining extracellular matrix integrity, and while they have also been linked to intracellular activities, decreases in the secreted levels of TIMP-3 and PAI-2 were observed [129]. These findings suggest that negative transcriptional regulation of protease inhibitors by ΔNp63α may contribute to a more permissive environment for tumor invasion. Additional support for a role of ΔNp63α in extracellular matrix remodeling was derived from breast cancer models. ΔNp63α was shown to positively regulate membrane-type 1-matrix metalloproteinase (MT1-MMP) in breast cancer cells through direct promoter binding and overexpressing ΔNp63α in this context correlated with the ability to invade a 3D matrix of type 1 collagen [130]. Notably, in a 3D organoid model of breast cancer, activation of a basal epithelial gene program marked by keratin 14 and p63 induced invasive behavior dependent on collagen 1 [131]. Whether similar requirements occur in squamous cancers is not yet established; however, taken together, these data suggest that increased expression of ΔNp63α in tumor cells may facilitate stromal invasion. In the skin setting, silencing of ΔNp63α in p38α knockout mouse keratinocytes restored matrix metalloproteinase (MMP) 13 expression; significantly, MMP13 knockout mice presented with an increased incidence of tumors relative to WT controls [132]. This study revealed a potential role for MMP13 as a tumor suppressor [133], with its expression inhibited by ΔNp63α [132]. ΔNp63α has also been implicated as an angiogenic factor in SCC. In human keratinocytes and several SCC cell lines, ΔNp63 was shown to induce tumor angiogenesis and lymphangiogenesis via activating human beta-defensins [134]. Additional studies are needed to further clarify the role of ΔNp63α in extracellular matrix remodeling and the implications for cancer cell migration and invasion.

Increasing evidence indicates that ΔNp63α influences the tumor immune microenvironment. In human epithelial cells, including the HaCaT epidermal and Lc817 lung cancer cell lines, p63 positively regulates TARC/CCL17, a ligand of CCR4 that acts as a T-cell chemoattractant [135]. The discovery that elevated ΔNp63α leads to activation of NF-κB/c-Rel, a known mediator of inflammatory responses [32], along with the correlation of c-Rel in HNSCC cancer cells harboring high p63 [32,136] levels and the expanded nuclear expression of these proteins in human HNSCC specimen, suggested that coordinated gene regulation by ΔNp63α and c-Rel might explain the heavy immune cell infiltrate typically seen in these poorly responsive SCC [136,137]. These observations were reiterated in the skin of keratin 5-Cre-targeted ΔNp63α transgenic mice, which display a hyperproliferative epidermis with suprabasal expression of ΔNp63α and corresponding enhanced nuclear localization of c-Rel, as well as an inflammatory dermal infiltrate of lymphoid and myeloid lineages [136,137,138]. Indeed, gene profiling of the skin of the ΔNp63α transgenics revealed that 19% of the genes observed to be upregulated were related to inflammatory and immune responses, and a subset of these are co-regulated by ΔNp63α and NF-κB [137]. Thus, ΔNp63α contributes to the regulation of proinflammatory cytokines and chemokines, thereby shaping the tumor immune microenvironment. Additional evidence of a role for ΔNp63α in tumor-associated inflammation and immune evasion was recently reported in a xenograft model of triple negative breast cancer, in which ΔNp63 was found to recruit myeloid-derived suppressor cells, indicating a role in immune evasion [139]. The promise of leveraging the growing knowledge regarding this network to reactivate the immune system for detecting and eliminating tumors is seen in the expanding clinical applications of checkpoint inhibitors in SCC [140].

## 5. Conclusions

ΔNp63α is a key regulator of epidermal morphogenesis and epithelial tissue homeostasis. In addition to direct targeting of gene transcription, p63 functions as a key driver of critical global networks linked to cellular identity and cell fate determination. Dysregulated expression of p63 is a common feature of squamous cancers arising across organ sites and is believed to contribute to cancer development through disruption of numerous cellular processes (Figure 3). In addition to influencing keratinocyte lineage commitment, proliferation and survival, ΔNp63α can modulate the tissue microenvironment, recruiting immune components and potentially altering the balance between immune surveillance and immune evasion. As the understanding of cooperative interactions between p63 and coordinated pathways is expanded and new mechanisms of gene regulation by p63 are uncovered, deciphering and manipulating the key players hold promise for novel interventions for cancer prevention and treatment.

## Figures and Tables

**Table 1:** Dissecting the role of ΔNp63α in development and cancer – murine models.

Role	Model	Epidermal Phenotype	Reference
**Morphogenesis/Stratification/** **Homeostasis: Genetic Models**	*p63^−/−^*	Lack of complete stratified epithelium, absence of keratin 5 or keratin 14	[18,19]
	Keratin 5-ΔNp63α complementation (*p63^−/−^* background)	Greater degree of epithelialization and greater amounts of keratin 5 and keratin 14 expression relative to p63^−/−^	[51]
	Keratin 5-TAp63α and keratin 5ΔNp63α complementation (*p63^−/−^* background)	Greatest degree of organized epithelialization relative to both single complementation models (TA or ΔN)	[51]
	Tet-keratin 5-ΔNp63α or Tet-keratin 5-ΔNp63β (*p63^−/−^* background)	Partial restoration of epidermal integrity with focal expression of keratin 5, keratin 1, and filaggrin	[52]
	*ΔNp63α^−/−^* (exon replaced with GFP/GFP)	Lack of complete stratified epidermis; dysregulated basal keratin expression	[53]
	Keratin 5-Cre mediated *p63* ablation	Increased cellular senescence marker expression Embryonic: loss of stratified squamous epithelium; lack of keratins 14, 1, and 10 and filaggrin Adult: epidermal defects	[58]
**Tumor Development and Progression:** **ΔNp63** **α Overexpression Models**	*p53^+/−^p63^+/−^*	Higher frequency of squamous cell carcinomas (of various organ sites) and metastatic tumors relative to p53^+/−^	[57]
	*p63^+/−^*	Squamous cell hyperplasia; increased number of spontaneous tumors (including squamous cell carcinoma, organ site not specified)	[57]
	Keratin 5-ΔNp63α	Increased susceptibility to chemical carcinogenesis	[60]
	Orthotopic grafting of primary murine keratinocytes expressing oncogenic Ras and elevated ΔNp63α	Malignant conversion of keratinocytes in vivo; inhibition of cellular senescence (reduced p16 and p19 levels)	[90]
	Subcutaneous engraftment of primary murine keratinocytes expressing oncogenic Ras and elevated ΔNp63α	Squamous cell carcinomas; inhibition of cellular senescence (increased Lsh expression)	[92]
	Conditional deletion of p63 in p53-deficient mice (*p63^L/L^K14-CreER/p53^+/−^*)	Regression of carcinogen-induced tumors	[95]

## Abbreviations

TAD	transactivation domain
DBD	DNA binding domain
OD	oligomerization domain
SAM	sterile alpha motif
TID	transcriptional inhibitory domain
WT	wild type
HNSCC	head and neck squamous cell carcinoma
miRNA	microRNA
lncRNA	long non-coding RNA
SA-β-gal	senescence-associated β-galactosidase
HDAC	histone deacetylase
BMP	bone morphogenetic protein
SHH	sonic hedgehog
FGFR2	fibroblast growth factor receptor 2
TGF-β	transforming growth factor β
EDC	epidermal differentiation complex
EEC	Ectrodactyly Ectodermal Dysplasia-Clefting Syndrome
AEC	Ankyloblepharon-Ectodermal Dysplasia Clefting Syndrome
SCC	squamous cell carcinoma
TCGA	The Cancer Genome Atlas
cSCC	cutaneous squamous cell carcinoma
ChIP-Seq	chromatin immunoprecipitation-sequencing
EGFR	epidermal growth factor receptor
MAPK	mitogen-activated protein kinase
FGF	fibroblast growth factor
EMT	epithelial-mesenchymal transition
iASPP	inhibitor of apoptosis-stimulating protein of p53
CSC	cancer stem cell
HK2	hexokinase 2
ROS	reactive oxygen species
GPX2	glutathione peroxidase 2
PAI-2	plasminogen activator inhibitor-2
TIMP	tissue inhibitor of metalloproteinase
MT1-MMP	membrane-type 1-matrix metalloproteinase
MMP	matrix metalloproteinase
